# Molecular Landscape and Treatment Paradigms of Hepatocellular and Cholangiocarcinoma: A Multinational Review

**DOI:** 10.1055/a-2548-0108

**Published:** 2025-03-31

**Authors:** Kristijan Skok, Judith Stift, Peter Schirmacher, Karl Kashofer, Rudolf Stauber, Branislava Ranković, Karoline Lackner

**Affiliations:** 1 31475 Diagnostic and Research Institute of Pathology, Medical University of Graz, Graz, Austria; 2 68939 Insitute of Biomedical Sciences, Faculty of Medicine University of Maribor in Slovenia, Maribor, Slovenia; 3 Institute of Pathology, Versorgungspathologie of the University Clinic of Innsbruck, INNPATH GmbH, Innsbruck, Austria; 4 ADK Diagnostics, Center for Liver and Pancreatic Pathology, Vienna, Austria; 5 155995 Heidelberg University Hospital Institute of Pathology, Heidelberg, Germany; 6 31475 Internal Medicine, Medical University of Graz, Graz, Austria; 7 37664 Institute of Pathology, University of Ljubljana Faculty of Medicine, Ljubljana, Slovenia

**Keywords:** Hepatocellular Carcinoma, Cholangiocarcinoma, Molecular Targeted Therapy, Precision Medicine, Molecular Diagnostics, Hepatozelluläres Karzinom, Cholangiokarzinom, Präzisionsmedizin, Molekulare Diagnostik, Zielgerichtete Therapie

## Abstract

Hepatocellular carcinoma (HCC) and cholangiocarcinoma (CCA) represent the most prevalent primary liver cancers and pose significant challenges in oncology. While their etiology and incidence vary globally, the molecular landscape of these tumors is increasingly understood, offering new opportunities for precision medicine. In this joint multinational review, we present a comprehensive analysis of the key molecular pathways involved in the pathogenesis of HCC and CCA, highlighting actionable targets for emerging therapies. Recent advances in molecular diagnostics have significantly influenced treatment paradigms for both cancers. In HCC, while genetic alterations have not yet led to established diagnostic or therapeutic applications, targeting vascular endothelial growth factor (VEGF), immune checkpoints, and tyrosine kinase pathways has demonstrated considerable therapeutic potential. In CCA, genetic profiling has uncovered actionable alterations, such as FGFR2 fusions and IDH1 mutations, driving the development of targeted therapies. The growing complexity of precision oncology underscores the need for standardized molecular testing and streamlined diagnostic workflows to ensure timely and effective treatment. This review also emphasizes the importance of collaborative efforts between clinicians, pathologists, and oncologists to optimize outcomes. By synthesizing the latest molecular insights and treatment trends, this review provides a valuable resource to guide the personalized management of HCC and CCA.

## Zusammenfassung

Das hepatozelluläre Karzinom (HCC) und das Cholangiokarzinom (CCA) gehören zu den häufigsten primären Leberkarzinomen und stellen eine erhebliche onkologische Herausforderung dar. Während Ätiologie und Inzidenz weltweit unterschiedlich sind, liefert ein besseres Verständnis der molekularen Grundlagen dieser Tumoren neue Ansätze für die Präzisionsmedizin. In dieser gemeinsamen multinationalen Übersichtsarbeit analysieren wir die zentralen molekularen Signalwege, die an der Pathogenese von HCC und CCA beteiligt sind, und beleuchten gezielt nutzbare Therapieansätze. Jüngste Fortschritte in der molekularen Diagnostik haben die Behandlungsstrategien für beide Tumorarten verändert. Beim HCC haben genetische Alterationen bislang zwar noch keinen festen Platz in der Diagnostik oder Therapie, jedoch zeigt die gezielte Hemmung des vaskulären endothelialen Wachstumsfaktors (VEGF), von Immun-Checkpoints und Tyrosinkinase-Signalwegen vielversprechendes therapeutisches Potenzial. Beim CCA ermöglicht die genetische Analyse die Identifikation nutzbarer Mutationen wie FGFR2-Fusionen und IDH1-Mutationen, die gezielte Therapien vorantreiben. Die zunehmende Komplexität der Präzisionsonkologie erfordert standardisierte molekulare Tests und optimierte Diagnostikabläufe, um eine rechtzeitige und effektive Behandlung sicherzustellen. Diese Übersichtsarbeit betont die Bedeutung einer interdisziplinären Zusammenarbeit zwischen Klinikern, Pathologen und Onkologen, um Behandlungsergebnisse zu verbessern. Durch die Zusammenführung aktueller molekularer Erkenntnisse und Therapieentwicklungen bietet diese Übersichtsarbeit eine wertvolle Grundlage für die personalisierte Behandlung von HCC und CCA.

## Introduction 1

Hepatocellular carcinoma (HCC) is the most common primary liver tumor and one of the leading causes of cancer-related deaths worldwide. Its incidence varies significantly across different countries and regions. In Asia and Africa, the incidence is higher compared to Europe and North America. HCC is more commonly seen in older adults, particularly in men, with a male-to-female ratio often around 2–4:1. In 2022, more than 860,000 people worldwide were diagnosed with primary liver cancer (HCC and intrahepatic cholangiocarcinoma, iCCA), with over 750,000 deaths reported, according to GLOBOCAN data 1 . The incidence has been rising in recent years. Up to 80% of cases occur in Southeast Asia and sub-Saharan Africa, largely due to the high prevalence of chronic hepatitis B virus (HBV) infection. In Europe, North America, and Japan, HCC incidence is lower 2 . In Germany, the primary risk factors are chronic hepatitis C virus (HCV) infection and alcohol consumption 2 . In Western countries, HCC incidence has increased significantly due to liver cirrhosis from chronic HCV infection and the growing prevalence of metabolic dysfunction-associated steatotic liver disease (MASLD) and metabolic dysfunction-associated steatohepatitis (MASH) with advanced fibrosis or cirrhosis 3 . Liver cirrhosis remains the most significant risk factor for HCC development. Regardless of the underlying cause – HCV, HBV, MASH, alcohol abuse, hemochromatosis, α1-antitrypsin deficiency, or others – patients with cirrhosis have an elevated HCC risk. The relative risk varies depending on the etiology 2 . Most HCCs develop within so-called dysplastic nodules, which are partially associated with etiology and arise from the accumulation of clonal genetic alterations in regenerative nodules of the cirrhotic liver. The development of HCC from a hepatocellular adenoma is comparatively rare 4 5 .

CCA is a rarer primary malignant liver tumor compared to HCC, originating in the bile ducts. Epidemiological data indicate that CCA are more prevalent in certain geographic regions, particularly in Southeast Asia, due to the high prevalence of bile duct infestations by liver flukes 6 .

Other common risk factors include cirrhosis (caused by chronic liver diseases, similar to HCC) and chronic inflammation of the bile ducts, such as primary biliary cholangitis or primary sclerosing cholangitis 4 .

Although the molecular mechanisms underlying the development of primary liver carcinomas are not yet fully understood, several key pathophysiological mechanisms have been found, providing a foundation for the development of new individualized treatment options.

## Hepatocellular carcinoma

### Definition, Macroscopy and Histology

The HCC is a primary malignant liver tumor composed of epithelial cells showing hepatocellular differentiation 7 . Its macroscopic appearance is variable. HCC may present as a single well-demarcated nodule or as multiple nodules, such as one large nodule with adjacent smaller nodules, multiple small closely situated nodules, or several distinct nodules of similar size. The color of the tumor’s cut surface can range from yellow-brown (due to fat accumulation in tumor cells) to brown-green (tumor cells with bile production). The tumor cells exhibit a hepatocyte-like morphology and differentiation. They are usually smaller than non-neoplastic liver cells and are arranged in broad trabeculae consisting of several layers of cells, which are covered by non-fenestrated endothelial cells. HCC is highly vascularized, with sinusoid-like structures and occasionally small arterial blood vessels found between the endothelial-covered trabeculae.

**Fig. 1:**
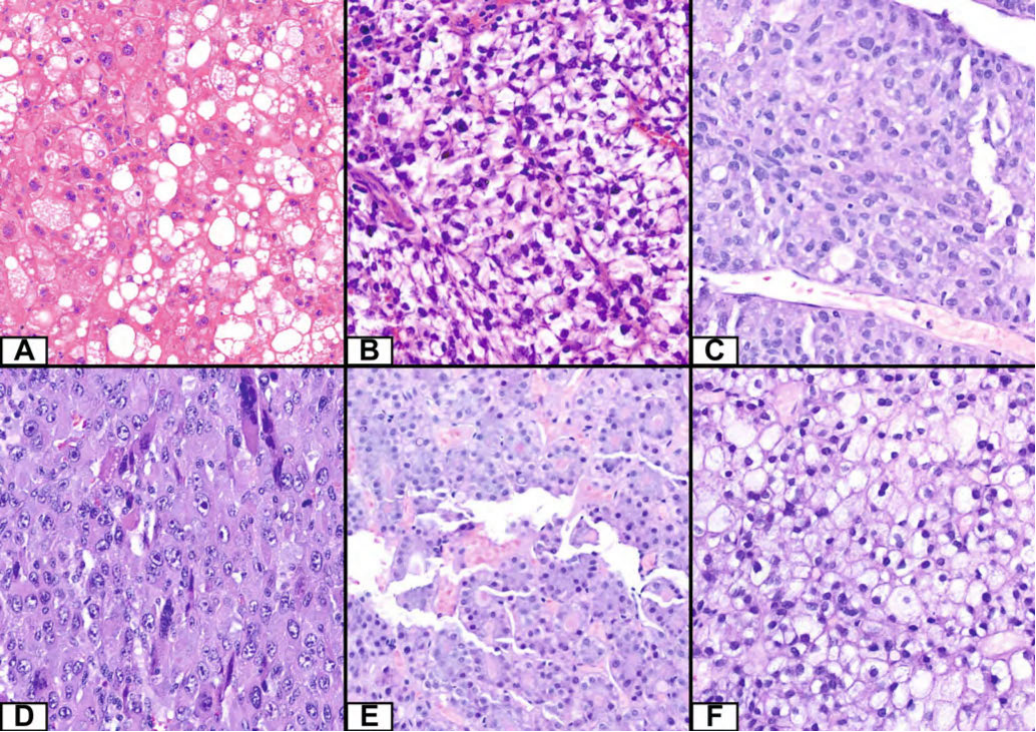
Some common histological subtypes and growth patterns of HCC. Legend: **A** – steatohepatitic subtype, **B** – clear cell subtype; **C** – macrotrabecular subtype, **D** – solid growth pattern, **E** – pseudoglandular growth pattern, **F** – solid growth pattern with fatty changes (40× Magnification; source – own).

**Fig. 2:**
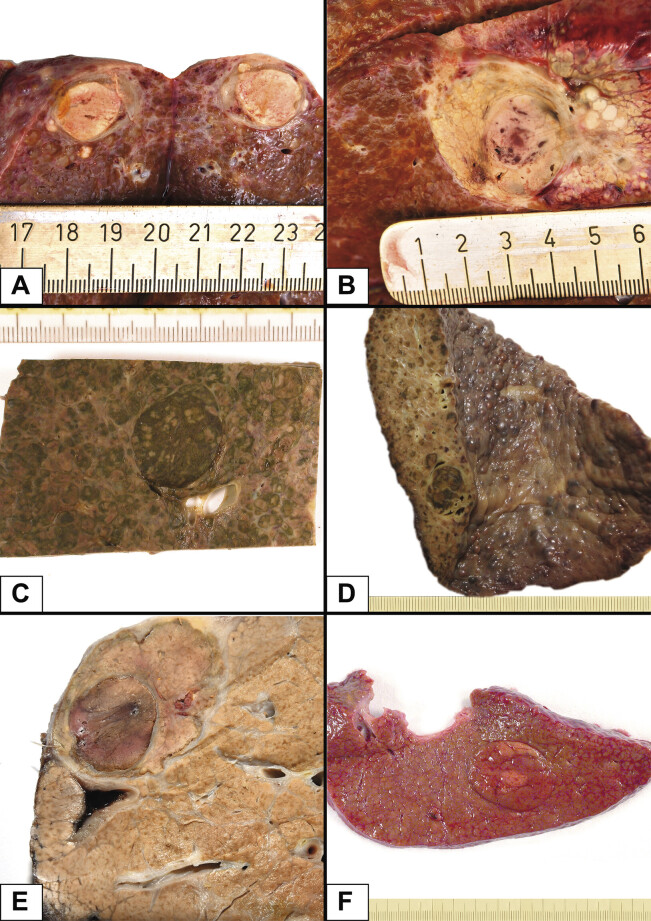
**A** and **B** – HCC in cirrhotic liver with a nodule-in-nodule growth and smaller satellite nodules; **C** and **D** – HCC in cirrhotic liver with cholestasis (a macroscopic and microscopic feature useful for distinguishing it from other carcinomas, i.e. metastasis or intrahepatic CCA); **E** and **F** – HCC in cirrhotic liver nodule-in-nodule growth.

Depending on the arrangement of the tumor cells, we can differentiate different histological types of HCC as well as four principal growth patterns. These patterns are trabecular, solid (synonym: compact), pseudoglandular (synonym; pseudoacinar), and macrotrabecular (composed mostly of trabeculae, being ≥ 10 cells thick). About 50% of resected HCCs have mixed patterns, usually trabecular plus one or two others ( Fig. 1 ) 7 . As many as 35% of HCCs can be further subclassified into distinct subtypes (see Table 1 ), representing distinct clinicopathological/molecular entities. For example, specific cellular changes (e.g., fat accumulation, bile production, glycogen storage, protein aggregates such as Mallory-Denk bodies, pale bodies, or globular hyaline inclusions) characterize further histological subtypes of HCC (e.g., steatohepatitic, clear-cell, macrotrabecular massive etc.) 8 . In larger tumors, a “nodule-in-nodule” growth pattern is often observed ( Fig. 2 ), where poorly differentiated tumor nodules arise within a well-differentiated nodule through clonal expansion. The differing levels of differentiation are often visible macroscopically as well. Interesting to note is the fact that HCCs can still produce bile, which is a very useful clue when diagnosing and differentiating tumor entities, be it macroscopically ( Fig. 2 C and D) or microscopically. Such a macroscopic feature can already tell the pathologist that the tumor cannot be a metastasis nor a CCA.

**Table:** **Table 1** Overview of the key characteristics of more common HCC subtypes.

Subtype	Frequency	Clinical properties	Histology	Molecular properties
Steatohepatitic	5–20%	Can be associated with Steatohepatitis	Steatotic changes, ballooning of the cells, inflammatory foci, “Mallory-Denk bodies”	IL-6 / JAK / STAT activation; low frequency of CTNNB1, TERT, and TP53 mutations
Clear-cell	3–7%	Not known	>80% of tumor cells with clear cell morphology, potentially mild steatotic changes	Not known
Macrotrabecular	5%	High serum AFP, poor prognosis:	Macrotrabecular growth in >50% of the tumor, vascular invasion is common	TP53 mutation FGF19 amplification
Scirrhous	4%	Can resemble CC	>50% of the tumor with dense intratumoral fibrosis	TSC1/2 mutation; “TGFbeta-signalling”
Chromophobe	3%	Not known	Tumor cells with chromophobic cytoplasm, inconspicuous nuclei, areas with anaplastic changes and microcysts	Alternative telomere elongation
Fibrolamellar	1%	Young, no other liver diseases	Large oncocytic tumor cells (K7 and CD68 positive) with prominent nucleoli	DNAJB1-PRKACA fusion gene
Neutrophil-rich	<1%	Leukocytosis, increased CRP and IL-6, poor prognosis	Prominent infiltration of polymorphic granulocytes, sarcomatoid areas may be present	G-CSF expression by tumor cells
Lymphocyte-rich	<1%	Not known	More lymphocytes than tumor cells	Not known, EBV independent
Legend: AFP – alpha-fetoprotein, EBV – Epstein-Barr virus, CC – clear-cell, IL-6 – interleukin 6, JAK – Janus Kinase, STAT – Signal Transducer and Activator of Transcription, TERT – Telomerase Reverse Transcriptase, CTNNB1 – Catenin Beta 1, FGF19 – Fibroblast Growth Factor 19, TSC1/2 – Tuberous Sclerosis Complex 1 and 2, K7 – keratin 7, CD68 – Cluster of Differentiation 68, DNAJB1-PRKACA – DNA J Binding Protein 1 – Protein Kinase A Catalytic Subunit Alpha, CRP – C-reactive Protein, G-CSF – Granulocyte Colony-Stimulating Factor. Adapted from WHO 5. Edition (Paradis V, Fukayama M, Park YN, Schirmacher P, World Health Organization (ed) digestive system tumours: WHO classification of tumours, 5 ^th^ Edition, Volume 1. Tumours of the liver and intrahepatic bile ducts, Lyon 219, 215–264, 5 ^th^ Edition ISBN 978–92–832–4499–8) and Longerich et al. 9				

### Diagnosis and therapy

The high vascularity of HCC forms the basis for its non-invasive contrast-enhanced radiological diagnosis in cirrhotic livers using CT or MRI (with liver-specific contrast agents). Furthermore, studies indicate that contrast-enhanced ultrasound (CEUS) is suitable for monitoring HCC in at-risk patients, offering high sensitivity and specificity. Advantages of CEUS include rapid availability, no nephrotoxicity, no restrictions in thyroid dysfunction, and fewer contrast medium allergic reactions. In cases of unclear MRI findings or contraindications to MRI, a triphasic CT (late arterial/portal venous/late venous phase) or CEUS should be used for further diagnostics 2 10 11 .

In many cases of cirrhosis, histological examination is not required but it is always indicated for tumors in a non-cirrhotic liver. The classification of HCC should align with the latest WHO guidelines. This involves differentiating special types (such as fibrolamellar HCC and mixed-differentiated tumors like combined HCC/iCCA), and, where possible, distinguishing early-stage HCC from progressive HCC and pre-cancerous lesions. It is also important to clearly differentiate between rare forms of iCCA, liver metastases, and benign liver tumors. The same applies to surgical specimens, where the TNM classification, typing, grading, resection margin evaluation and description of the non-neoplastic liver must be done. If conventional histology is not sufficient, additional diagnostic modalities can be applied (molecular testing, immunohistochemistry etc.) 2 .

The complication rate of HCC biopsy is low, with minor bleeding occurring in 3–4% of cases and transfusion-requiring bleeding in 0.5%. Needle track seeding is rare (2.7%), typically occurring after 17 months, and is usually treatable 12 13 .

The treatment of HCC depends on the size and number of tumors, radiologically detected vascular invasion, and clinical parameters (such as the patient’s general condition and liver function). It is often guided by a diagnostic-therapeutic algorithm, such as the Barcelona Clinic Liver Cancer (BCLC) classification 14 .

### Molecular Landscape and Subtypes

Genetic alterations in proto-oncogenes, tumor suppressor genes, and genes involved in the cell proliferation cycle, apoptosis, and cell differentiation play a significant role in the development of HCC. These changes include mutations, amplifications, deletions, insertions, and translocations, leading to the overexpression of oncogenes and the progression of cancer. Notably, insertion mutations frequently occur in HBV-associated HCC 15 .

HCC can be classified into two main molecular classes based on transcriptomic phenotypes: the proliferation class and the non-proliferation class 16 17 . The proliferation class includes more aggressive tumors with poor differentiation, increased vascular invasion, and elevated AFP levels 8 18 . This group accounts for nearly half of all HCC cases and is often associated with HBV infection. The most common genetic changes in this class are TP53 mutations, amplifications of FGF19 and CCND1, and increased chromosomal instability 19 20 . The tumors of the proliferation class can be further divided into two subclasses: the proliferation-progenitor cell subclass and the proliferation-Wnt-TGF-β subclass.

The non-proliferative tumor class is characterized by less aggressive, well to moderately differentiated tumors, often associated with metabolic-dysfunction associated steatohepatitis (MASH), HCV infections, and low AFP levels. This class can be subdivided into two specific subclasses: the Wnt-β-catenin-CTNNB1 subclass and the interferon subclass. The former frequently exhibits CTNNB1 mutations and is characterized by the activation of the Wnt-β-catenin signalling pathway 19 20 21 . TERT promoter mutations are common in this subclass. The interferon subclass is characterized by an activated IL-6-JAK-STAT signalling pathway 20 .

HCCs with mutations in the CTNNB1 gene (CT-HCC) exhibit a distinct phenotype with well-differentiated tumors, microtrabecular and pseudoglandular architectural patterns, intratumoral cholestasis, and absent or low lymphocytic infiltration 16 17 . However, these features are not specific to CT-HCC, as they can be found in almost 40% of all HCC cases 19 . Moreover, some studies have shown that the rate of CTNNB1 mutations is significantly lower in HBV-associated HCC compared to other etiologies 22 .

The TP53 gene is one of the most important tumor suppressor genes. The p53 protein encoded by TP53 is involved in various signalling pathways to regulate multiple processes, including metabolism, DNA repair, cell cycle arrest, and apoptosis. As in other cancers, TP53 mutations are also one of the main genetic alterations in HCC, occurring in about 30% of HCC cases. Inactivating TP53 mutations contribute to the initiation and progression of HCC 23 . CT-HCC and HCC with TP53 mutations do not occur together.

Another crucial driver mutation in HCC carcinogenesis involves mutations in the promoter region of the TERT gene, leading to the activation of the telomerase complex and thus the elongation of telomeres. The telomerase complex consists of telomerase reverse transcriptase (TERT), the telomerase RNA component (TERC), and several proteins such as the shelterin components TRF1, TRF2, TIN2, RAP1, TPP1, and POT1. The liver shows low physiological telomerase expression 24 . During chronic liver injury and inflammation, hepatocytes experience progressive telomere shortening. Without telomerase activity, this can lead to chromosomal erosion and genomic instability, which is often mitigated by p53-induced senescence and, in severe cases, apoptosis 25 26 . Telomerase is primarily reactivated through the following mechanisms: mutations in the promoter region of the TERT gene (54–65% of HCCs), TERT gene amplification (5–6%), and TERT gene translocations (2–3%) 27 . Telomerase is an attractive target for selective cancer therapy due to its crucial role in enabling cell immortality and its significant involvement in the progression of liver tumors. TERT mutations are widespread and detectable in up to 90% of HCC patients 24 .

Epigenetics encompasses all processes where the activity of a gene is altered without changing its DNA sequence, and this alteration is passed on to daughter cells. Epigenetic dysregulation, including changes in DNA methylation, abnormalities in histone deacetylation, chromatin remodelling, and dysregulated expression of long non-coding RNAs (lncRNAs) and microRNAs (miRNAs), is observed in 20–50% of HCC cases. Methylation changes often accompany the initiation and progression of HCC, influenced by HBV, HCV, and other risk factors 15 .

miRNAs are small non-coding RNA molecules that act as epigenetic gene regulators. In association with the RNA-induced silencing complex (RISC), miRNA and RISC can bind to complementary mRNA sequences, leading to post-transcriptional degradation or downregulation of specific gene activities 28 . Numerous studies have highlighted altered levels of specific miRNAs in various cancers, including HCC. Since miRNAs can be both upregulated and downregulated, their dysregulation impacts a variety of signalling pathways that often modulate cell proliferation, differentiation, migration, and survival. For example, miR-144 is believed to be a tumor suppressor that is downregulated during tumor progression. Similarly, the tumor suppressor miR-342–3p is elevated in HCC but reduced during tumor regression 26 27 . Other tumor suppressor miRNAs, such as miR-1, miR-124, miR-214, miR-34a, and miR-449, target mRNA molecules involved in tumor progression and are generally downregulated in HCC 29 .

lncRNAs are another class of RNA molecules that regulate gene expression post-transcriptionally. Most of these lncRNAs are transcribed at low levels, making them difficult to detect. However, a change in the lncRNA signature, measured in blood, can indicate a tumor process in the liver. The analysis of the Cancer Genome Atlas (TCGA) shows that the upregulation of the lncRNA LINC01234 is associated with a poor prognosis in HCC patients 26 30 . Some lncRNAs may regulate gene expression by suppressing the expression of miRNAs.

Determining molecular profiles can distinguish rare forms of HCC and be helpful in cases where morphology is insufficient for a definitive diagnosis. Fibrolamellar carcinoma (FLC) is a rarer subtype of HCC that typically occurs in younger patients and is not associated with the previously mentioned etiologies. FLC can be confused with the cirrhotic type of HCC. Recent studies have identified genetic variations that distinguish FLC from normal liver parenchyma and conventional HCC. Notably, Honeyman et al. discovered a 400 kb deletion on chromosome 19, present in all FLC tumors, leading to a DNAJB1-PRKACA chimeric transcript that further defines FLC as a distinct entity 31 . FLCs also exhibit abnormal methylation patterns of tumor suppressor genes in promoter regions, similar to the common hypermethylation seen in conventional hepatocellular carcinomas 19 . One of the most important differential diagnostic considerations is tumors with mutations in the BAP1 gene and activation of protein kinase A – a rare type of HCC showing features similar to fibrolamellar carcinoma, BP-HCC 32 . This subtype typically manifests in older patients and is associated with a poorer prognosis.

### Targeted therapy as a new individualized treatment option in HCC

Although the landscape of genetic alterations in HCC can increasingly be characterized with greater precision, these findings are not yet used in the diagnosis of HCC. However, based on the knowledge of molecular genetic changes in HCC and other tumor entities, new therapeutic strategies have been developed (see below), which primarily target tumor cells and affect non-neoplastic cells less (known as targeted therapy). These drugs are currently used in the treatment of advanced HCC (also known as advanced-stage HCC; see BCLC classification) 4 . Some signalling pathways are associated with the development of HCC and thus serve as potential therapeutic approaches. These include the Ras/Raf/MAPK, PI3K/Akt/mTOR, Wnt/β-Catenin, JAK/STAT, Hippo-YAP/TAZ, Hedgehog, and Notch signalling pathways 15 .

HCC is characterized by high expression of angiogenic promoters (Angiogenin 2, PDGF, and VEGF). Current strategies for molecular targeted therapies in HCC mainly focus on VEGF. In addition to sorafenib, which was for a decade the only available first-line standard treatment for advanced HCC, new first-line therapeutics have been introduced. Bevacizumab is a monoclonal antibody against VEGF-A. Increasing data support the use of bevacizumab in combination with atezolizumab, a humanized monoclonal antibody that acts as an immune checkpoint inhibitor, as first-line therapy for the treatment of advanced HCC 33 . The combination of tyrosine kinase inhibitors or VEGF inhibitors with immune checkpoint inhibitors can modulate the immune microenvironment by enhancing dendritic cells (DCs) and cytotoxic T lymphocytes, while inhibiting tumor-associated macrophages (TAMs), regulatory T cells (Tregs), and myeloid-derived suppressor cells (MDSCs). This creates a more inflammatory microenvironment that favors the development of more effective and long-lasting responses to checkpoint inhibitors 34 35 .

Patients respond differently to targeted therapies ( Table 2 **)** . Currently, biomarkers are being developed to predict the efficacy of treatment, in order to identify patients who are most likely to respond to the therapy and spare those who do not respond to the side effects of an ineffective treatment 15 . In addition, ongoing studies investigate whether circulating miRNAs and lncRNAs, which are easily accessible through a blood test (“liquid biopsy”), can be used as biomarkers for the early detection of HCC 26 .

**Table:** **Table 2** Predictive biomarkers for the efficacy of targeted therapy in HCC.

Drug	Biomarker	Correlation
**Predictive biomarkers for the efficacy of tyrosine kinase inhibitors**		
Sorafenib	VEGF, VEGFR, HIF-1, eNOS.	Gene polymorphism
	VEGF-Response, Tumor-VEGFR, Amphiregulin-response, IGF, cytokine levels (IL-5, IL-8, TGF-α, PDGF-BB, CXCL9 and VEGF-A), pERK, FGF3/FGF4, ACSL4	Positive correlation
	Serum angiogenesis marker (Ang-2, HGF, G-CSF, Leptin), p-Met, ORM1.	Negative correlation
	Clinical predictors	
	Hypertension, diarrhea after sorafenib use, cutaneous adverse events within 60 days due to sorafenib usage, HCV, VETC.	Positive correlation
	Baseline-AST and NLR, FDG-uptake in PET, extrahepatic spread.	Negative correlation
Lenvatinib	FGF19, FGF19-reaction (increase), Ang-2-Reaction (decrease), ST6GAL1, Tumor-FGFR4.	Positive correlation
	VEGF, Ang-2, FGF21.	Negative correlation
	Clinical predictors	
	AFP-Reaction, ALBI-Score, Change in the ALBI-score, CEUS.	Positive correlation
Other	Cabozantinib: p-Met	Positive correlation
	Regorafenib: Ang-1, Cystatin B, LAP TGF-β1, LOX-1, MIP-1α	Negative correlation
**Predictive biomarkers for the efficacy of anti-angiogenic drugs**		
Bevacizumab	VEGFR2, Tregs and myeloid nflammation signatures in tumor tissues.	Positive correlation
Ramucirumab	AFP in concentrations of 400 ng/mL or greater.	Positive correlation
Adapted from Wang Y. and Deng B. 15 .		

Interesting to note is the study from Limousin et al. in which the authors describe molecular-based targeted therapies in patients with HCC and hepato-cholangiocarcinoma refractory to atezolizumab/bevacizumab 36 . The “French Medicine Genomic program 2025” has been designed to give patients with cancers that are refractory to systemic treatments access to off-label therapies adapted to their specific genomic profile. The authors have done whole-genome/-exome and RNA sequencing in all above-mentioned patients. Among 135 patients with HCC and H-CCK treated by atezolizumab/bevacizumab, 20 patients benefited from genomic analysis after progression 36 .

Nevertheless, it must be stated that thus far, no standalone genetic alteration can be used diagnostically or therapeutically in HCC treatment and the current treatment algorithms do not rely on angiogenic or any other molecular properties (see BCLC algorithm).

## Cholangiocarcinoma

### Definition, Macroscopy and Histology

**Fig. 3:**
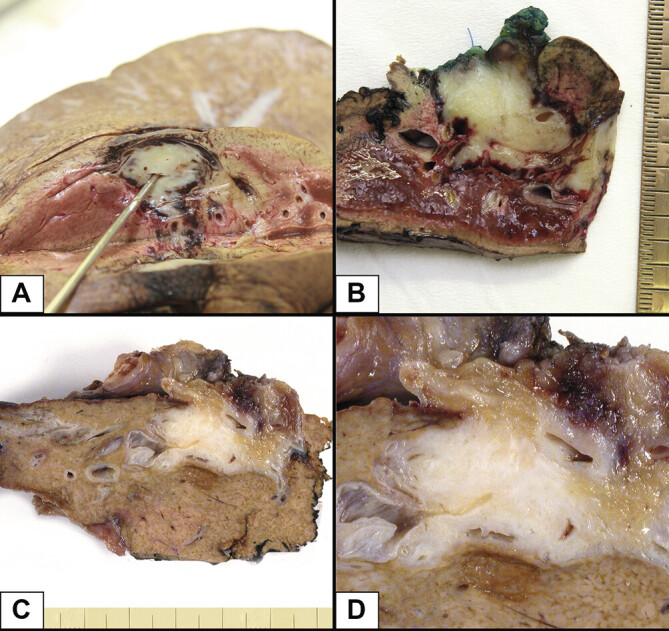
Macroscopic image of cholangiocarcinoma. Legend: **A** and **B** – same specimen; **C** and **D** – same specimen.

According to the recent (5 ^th^ edition) WHO classification of tumors of the digestive system, CCA are divided into intrahepatic cholangiocarcinomas (iCCA) ( Fig. 3 ) and perihilar as well as distal CCA, with the latter two often being subsumed as extrahepatic cholangiocarcinomas (eCCA). Gallbladder carcinomas are considered a separate entity 37 .

While it is assumed that the group of eCCAs arises from precursor lesions such as biliary intraepithelial neoplasia, the cellular origin of iCCA is still unclear and seems to be diverse. Experimental data from mice suggest that iCCA could originate from hepatocytes 38 . Intrahepatic CCA presents in a variety of morphological variants, but can generally be divided into two major groups, apart from the rarer forms: the “small-duct type” and the “large duct type” ( Table 3 ). Among the mentioned rare variants of iCCA are, for example, sarcomatoid iCCA and adenosquamous carcinoma (a mixed tumor consisting of adenocarcinoma and squamous cell carcinoma components) 39 40 .

**Table:** **Table 3** Subtypes of cholangiocarcinomas.

iCCA Subtypes	iCCA small duct Typ	iCCA large duct Typ
Risk factors	Chronic hepatitis B/C, MASH, generally chronic liver diseases.	PSC, gallstones, biliary helminthiasis ( *C. sinensis, O. viverrini* ),
Growth pattern	Nodule forming (mass-forming)	Periductal growth +/– mass forming; Intraductal growing
Precursor lesions	Precursor lesions are inadequately understood; among those discussed are ductal plate malformation and biliary adenofibroma.	Diverse epithelial forms of neoplasia (BilIN, IPNB, ITPN, MCN of the liver)
Histology	Cell-rich, with little stroma, no or very little mucin, coherently growing.	Fewer tumor cells compared to others, stromal-rich, (extracellular) mucin production; diffuse growth.
Common genetic alterations	Specific alterations as IDH1/2-mutations, FGFR2-fusion, BAP1-mutation;	Similar to eCCAs mutations in KRAS, p53, ARID1B, SMAD4
Legend: MASH – Metabolic Dysfunction-Associated Steatohepatitis; PSC – Primary sclerosing cholangitis, BilIN – biliary intraepithelial neoplasia, IPNB – Intraductal papillary neoplasm of the bile ducts, ITPN – Intraductal tubulopapillary neoplasm of the pancreas, MCN – Pancreatic mucinous cystic neoplasm; IDH1/2 – Isocitrate dehydrogenase 1 and 2; FGFR2 – Fibroblast growth factor receptor 2; BAP1 – BRCA1 associated protein-1; eCCAs – Extrahepatic cholangiocarcinoma; ARID1B – AT-rich interactive domain-containing protein 1B; SMAD4 – Suppressor of Mothers against Decapentaplegic 4. Adapted from WHO 5. Edition (Paradis V, Fukayama M, Park YN, Schirmacher P, World Health Organization (ed) digestive system tumours: WHO classification of tumours, 5 ^th^ Edition, Volume 1. Tumours of the liver and intrahepatic bile ducts, Lyon 219, 215–264, 5 ^th^ Edition ISBN 978–92–832–4499–8). 37 .		

**Fig. 4:**
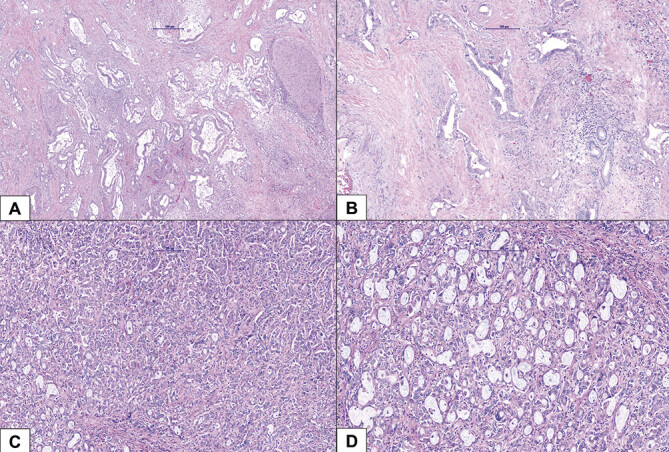
Different histologic subtypes of cholangiocarcinoma. Legend: **A** (20× magnification) and **B** (40× magnification) – large-duct-iCCA; **C** (20× magnification) and **D** (40× magnification) – small-duct-iCCA.

Morphologically, the large duct type ( Fig. 4 **)** usually arises in and around large intrahepatic bile ducts, where biliary neoplasms (BilIN), biliary intraductal papillary neoplasia (IPNB), and mucinous cystic neoplasia of the liver (MCN) are considered precursor lesions. This is not the case for small-duct iCCA. The growth form also differs from that of the small-duct types. In the large-duct type, there is often periductal or intraductal spread, and morphologically, it is mostly mucin-producing ductular, tubular, or even papillary adenocarcinoma formations with a frequently extensive desmoplastic stromal reaction. Small-duct iCCA primarily occurs in the peripheral liver, appears as a small tubular or acinar adenocarcinoma with a nodular, mass-forming growth pattern (so-called “mass-forming type”), which invades the liver parenchyma and produces little or no mucus. Precursor lesions are poorly understood and often not detectable.

Mucinous, signet ring cell, clear cell, sarcomatoid, squamous cell, adenosquamous, mucoepidermoid, and lymphoepithelioma-like tumors represent histological differentiations and are considered very rare variants 37 .

### Molecular Landscape and Subtypes

The histological diversity also reflects the high molecular heterogeneity of iCCAs and can likely be attributed to their different origin cells and pathogenesis. According to recent studies, more than 50% of iCCAs exhibit potentially treatable genetic alterations 41 42 43 44 ( Table 4 ).

**Table:** **Table 4** Molecular landscape of cCCA and iCCA.

Molecular changes	dCCA/pCCA	iCCA
ARID1A Mutation	5–10%	5–15%
BAP1 Mutation	0–5%	5–15%
BRAF V600E Mutation	0–2%	3–6%
CDKN2A/B Mutation	10–20%	10–15%
ELF3 Mutation	3–10%	1–2%
ERBB2 Mutation	2–5%	2–3%
FGFR2 Mutation	0%	15–30%
IDH1/2 Mutation	0–3%	10–20%
KRAS/NRAS Mutation	20–40%	10–20%
MSI-H	1–3%	1–2%
NRG1 Translocation	<1%	<1%
NRTK Translocation	1–3%	1–3%
PBRM1 Mutation	n.a.	10–17%
PRKACA/PRKACB Translocation (IPNB-associated)	1–3%	0%
SMAD4 Mutation	5–15%	2–10%
TP53 Mutation	20–40%	20–30%
Legend: pCCA – peripheral cholangiocarcinoma, dCCA – distal cholangiocarcinoma, iCCA – intrahepatic cholangiocarcinoma. Adapted from Longerich et al. 9 .		

Based on the ESMO and S3 guidelines 2 45 , molecular testing is recommended for patients with advanced CCAs, especially iCCAs with small duct histology, as they are enriched for actionable targets. Next-generation sequencing (NGS) panels covering multiple genes are preferred over single-gene testing and can be performed on formalin-fixed paraffin-embedded tissue or, if tissue is insufficient, cell-free circulating DNA (liquid biopsy). Current panels should include Isocitrate Dehydrogenase 1 (IDH1), HER2/neu (ERBB2), and BRAF for hotspot mutations, while Fibroblast Growth Factor Receptor 2 (FGFR2) and NTRK gene fusions are best detected at the RNA level, ideally using hybrid capture or anchored multiplex PCR. Microsatellite instability (MSI) status can be assessed by immunohistochemistry (IHC) for mismatch repair proteins (MLH1, MSH2, MSH6, PMS2) or DNA-based assays. Collaboration with a molecular pathologist or tumor board is advisable to optimize testing strategies 2 45 .

Furthermore, serum CA 19-9 is a nonspecific marker elevated in biliary tract cancers and other gastrointestinal diseases; while not diagnostic, high levels suggest poor prognosis and may help monitor treatment response. Notably, ~10% of individuals (Lewis antigen-negative) cannot produce CA 19-9, limiting its utility in follow-up for these patients 2 45 .

Summarized, some of the recommendations from the current ECMO guidelines (Biliary tract cancer: ESMO Clinical Practice Guideline for diagnosis, treatment and follow-up* – Annals of Oncology) are that: 1) core biopsy should be obtained for diagnostic pathology and molecular profiling before any nonsurgical treatment; 2) In patients with d/pCCA without extraductal metastasis, PTC- or ERCP-guided biopsies should be carried out to obtain adequate tissue for diagnostic pathology and molecular profiling; 3) Depending on location, EUS-guided FNA or FNB may be an option to obtain biopsies of enlarged regional nodes and to obtain a tumour biopsy if ERCP-guided biopsies are negative or inconclusive; 4) Molecular analysis is recommended in advanced disease considered suitable for systemic treatment; 5) Elevated CA 19-9 is associated with poorer prognosis and can be useful for assessing response to treatment 2 45 .

While eCCA and iCCA share some common mutations, such as TP53, BRCA1, BRCA2, PIK3CA, KRAS, SMAD4, ARID1A, or GNAS, others are particularly typical for small-duct iCCA. These include IDH1, IDH2, and BAP1 mutations, as well as translocations affecting FGFR2, NRG1, ALK, and NTRK1–3. Additionally, the frequency of molecular alterations differs between CCAs, with iCCAs more frequently exhibiting alterations that can be targeted for therapy compared to eCCAs. The large-duct type is more likely to have mutations in oncogenes and tumor suppressor genes, similar to eCCAs.

Thus, the alterations typical of small-duct iCCA can also be used to identify iCCA in cases of an unclear primary tumor (CUP) 9 41 46 47 48 49 .

### Treatment options based on the molecular profile

In recent years, significant progress has been made in the treatment of CCA. Immunotherapy has gained traction due to the results of the TOPAZ-1 study, which explored combination immunotherapy with durvalumab. This human monoclonal antibody, combined with cisplatin and gemcitabine, has received approval for first-line treatment in the EU. Based on the guidelines 2 45 , the recommendations (Biliary tract cancer: ESMO Clinical Practice Guideline for diagnosis, treatment and follow-up* – Annals of Oncology) regarding the therapy regime are as follows:

**First-line Treatment:**

- cisplatin + gemcitabine is the standard of care (SoC) for patients with a performance status (PS) 0–1.
- Adding durvalumab to this regimen should be considered.
- oxaliplatin can replace cisplatin in cases of renal impairment.
- gemcitabine monotherapy is an option for patients with PS 2.

**Second- and Later-line Treatment:**

- FOLFOX is the SoC after cisplatin/gemcitabine.
- ivosidenib is recommended for IDH1-mutant CCA after progression on ≥1 prior therapy.
- FGFR inhibitors are recommended for FGFR2 fusions after ≥1 prior therapy.
- pembrolizumab is recommended for MSI-H/dMMR tumors after prior therapy.
- dabrafenib + trametinib is recommended for BRAFV600E mutations after prior therapy.
- PARP inhibitors may be considered for BRCA1/2 or PALB2 mutations after platinum response.
- NTRK inhibitors are recommended for NTRK fusions after prior therapy.
- HER2-directed therapy can be considered for HER2 alterations after prior therapy.
- Follow-up during treatment: every 8–12 weeks with CT/MRI and CA 19-9/CEA if secreted.

**Supportive Care:**

- Biliary drainage is recommended in obstruction; metal stents preferred if life expectancy >3 months.
- Sepsis due to obstruction requires prompt treatment.
- Patient education on stent patency, symptoms, and infection signs is essential 2 45 .

Molecular characterization has opened up new treatment options ( Table 5 ) 50 . These targeted approaches are currently being used as second-line therapies 45 . Among the changes currently being clinically investigated and recommended by ESMO for testing in advanced CCA are IDH1 mutations, FGFR2 and NTRK fusions, microsatellite instability, HER2, BRAF, and BRCA1/2 mutations. Other potentially targetable alterations include IDH2, ARID1A, PIK3CA, and BAP1 mutations, as well as MET and NRG1 fusions (overview below).

**Table:** **Table 5** Molecular targets in cholangiocarcinoma.

Gene alterations	Frequency	Therapy	ESCAT
IDH1	1–18% (8–18% iCCA)	IDH1 inhibitors (e.g. Ivosidenib)	IA
FGFR2 Fusions	<10% (5–15% iCCA)	FGFR inhibitors (e.g. Pemigatinib, Infigratinib)	IB
FGFR2 Mutations	2 % (2% iCCA)	FGFR inhibitors (e.g. Derazantinib, Erdafinib)	IIb
Her2 Amplifications	5%–10% (10%–20% dCCA, pCCA, GBC)	Anti-HER2 antibodies (e.g. Pertuzumab, Trastuzumab)	IIIA
Her2 Mutations	3%–5% (more common in dCCA, pCCA, GBC)	Anti-HER2 antibodies (e.g. Trastuzumab)	IIIA
BRAF Mutations	<5% (50% V600E)	BRAF inhibitors (e.g. Oabrafenib) and MEK inhibitors (e.g. Trametinib)	IB
NTRK Fusions	<1%	NTRK inhibitors (e.g. Larotrectinib, Entrectinib)	IC
BRCA1/2	3–5%	PARP inhibitors (e.g. Olaparib)	IIB / IIIA
MSI	<1%	ICIs (e.g. Pembrolizumab)	IC
PALB2	1%	First line Platin-based therapy, Second line PARP-Inhibitor (e.g. Rucaparib)	IIB
BAP1	5–15%	PARP inhibitor, EZH2 inhibitor	IIB
IDH2	<5% (<5% iCCA)	IDH2 inhibitors (e.g. Enasidenib)	
MET		Crizotinib	IV
NRG1	<1%	Afatinib / Erlotinib/Pertuzumab	IVA
ARID1A	5–15%	PARP inhibitor	IVA
Legend: ESCAT – ESMO Scale for Clinical Actionability of Molecular Targets; pCCA – peripheral cholangiocarcinoma, dCCA – distal cholangiocarcinoma, iCCA – intrahepatic cholangiocarcinoma; GBC- gallbladder carcinoma; ICI – immune checkpoint inhibitor. Adapted from the ESMO Clinical Practice Guideline 45 .			

#### IDH1– ivosidenib

IDH1 mutations are particularly common in iCCA. The reported prevalence varies significantly by country, with ESMO guidelines suggesting 8–18%, while other sources report 15–20%. Ivosidenib has been approved by the European Medicines Agency (EMA) and the U.S. Food and Drug Administration (FDA) for the treatment of adults with pretreated, locally advanced or metastatic CCA with an IHD1 mutation.

#### FGFR2-Fusion – pemigatinib, infigratinib, futibatinib

For FGFR2 fusions, two selective FGFR2 inhibitors and one covalent FGFR1–4 inhibitor (futibatinib) have already been approved. FGFR2 fusions occur in up to 15% of patients with iCCA.

#### HER-2 – trastuzumab deruxtecan, trastuzumab/pertuzumab, zanidatamab

ERBB2 alterations (amplification or mutation) are more frequently found in extrahepatic CCAs, especially in gallbladder carcinomas. Only about 3–4% of iCCAs are mutated, amplified, or overexpressed. For HER2 amplifications (HER2-positive iCCAs), the current ESMO guidelines recommend the combination of trastuzumab and pertuzumab. Zanidatamab is considered a promising substance based on current studies, with FDA approval expected in 2024.

#### BRAF-V600E – dabrafenib/trametinib

Just under 5% of iCCAs exhibit a BRAF mutation, about half of which are BRAF-V600E mutations and can be treated with targeted BRAF and MEK inhibitor therapy.

#### Microsatellite instability – pembrolizumab

For tumors with deficient mismatch repair (dMMR) or high-frequency MSI-H – and that have not received durvalumab in first-line therapy – treatment with pembrolizumab is recommended based on the KEYNOTE-158 study 45 .

#### NTRK-gene fusions – larotrectinib, entrectinib

Two substances, larotrectinib and entrectinib, have been approved by both the FDA and EMA for NTRK gene fusions, irrespective of tumor type. However, the proportion of CCAs with NTRK fusions is less than 1% of iCCAs.

## Summary

Molecular diagnostics are facing growing demands due to the increasing number of approved medications requiring predictive tests, new clinical approaches such as antibody-drug conjugates, and targeted therapies in adjuvant/neoadjuvant treatment. The need for molecular tests will continue to rise, especially as new tumor subtypes are identified. To adapt, diagnostic methods must develop standardized “one-size-fits-all” approaches to efficiently utilize time, resources, and materials. Personalized oncology approaches require timely testing and treatment, supported by infrastructures like specialized centers, to promote precision oncology and reduce failure rates in advanced tumor stages. A collaboration between clinical practice, oncology, and pathology is essential for this.

## Footnotes

1
A small, condensed part of this topic (less than a third of its total content) has been submitted as a professional article / small opinion piece to *SPECTRUM Onkologie* (no impact factor) in a different language (German language). The editorial board of *SPECTRUM Onkologie* has granted permission for the further use of the text in an indexed journal. The editorial board of ZfG has also been notified.
